# A role of right middle frontal gyrus in reorienting of attention: a case study

**DOI:** 10.3389/fnsys.2015.00023

**Published:** 2015-03-03

**Authors:** Shruti Japee, Kelsey Holiday, Maureen D. Satyshur, Ikuko Mukai, Leslie G. Ungerleider

**Affiliations:** ^1^Lab of Brain and Cognition, National Institute of Mental Health, National Institutes of HealthBethesda, MD, USA; ^2^Feinberg School of Medicine, Northwestern UniversityChicago, IL, USA; ^3^Laureate Institute for Brain ResearchTulsa, OK, USA

**Keywords:** right middle frontal gyrus, dorsal attention network, ventral attention network, resting state fMRI, endogenous attention, exogenous attention, reorienting of attention, frontal lobe tumor resection

## Abstract

The right middle fontal gyrus (MFG) has been proposed to be a site of convergence of the dorsal and ventral attention networks, by serving as a circuit-breaker to interrupt ongoing endogenous attentional processes in the dorsal network and reorient attention to an exogenous stimulus. Here, we probed the contribution of the right MFG to both endogenous and exogenous attention by comparing performance on an orientation discrimination task of a patient with a right MFG resection and a group of healthy controls. On endogenously cued trials, participants were shown a central cue that predicted with 90% accuracy the location of a subsequent peri-threshold Gabor patch stimulus. On exogenously cued trials, a cue appeared briefly at one of two peripheral locations, followed by a variable inter-stimulus interval (ISI; range 0–700 ms) and a Gabor patch in the same or opposite location as the cue. Behavioral data showed that for endogenous, and short ISI exogenous trials, valid cues facilitated responses compared to invalid cues, for both the patient and controls. However, at long ISIs, the patient exhibited difficulty in reverting to top-down attentional control, once the facilitatory effect of the exogenous cue had dissipated. When explicitly cued during long ISIs to attend to both stimulus locations, the patient was able to engage successfully in top-down control. This result indicates that the right MFG may play an important role in reorienting attention from exogenous to endogenous attentional control. Resting state fMRI data revealed that the right superior parietal lobule and right orbitofrontal cortex, showed significantly higher correlations with a left MFG seed region (a region tightly coupled with the right MFG in controls) in the patient relative to controls. We hypothesize that this paradoxical increase in cortical coupling represents a compensatory mechanism in the patient to offset the loss of function of the resected tissue in right prefrontal cortex.

## Introduction

In order to properly function in the world, individuals must be able to efficiently select information around them that is behaviorally relevant, and direct their attention toward it. Much has been written about how the human brain pays attention to stimuli in its environment. Previous research has identified two main neural mechanisms of visual attention: a top-down system, which acts in a goal-directed endogenous manner (e.g., Bundesen, 1990; Desimone and Duncan, 1995), and a bottom-up system, which responds to unexpected stimuli in an involuntary, stimulus-driven exogenous manner (e.g., Corbetta et al., 2000). It is well known that top-down control of visual attention can be localized to a dorsal frontoparietal network of brain regions, including bilateral frontal eye fields (FEF), superior parietal lobule (SPL), and intraparietal sulcus (IPS) (e.g., Corbetta and Shulman, 1998; Kastner et al., 1999; Hopfinger et al., 2000, see Chica et al., 2013 for review). This network of regions is often referred to as the Dorsal Attention Network (DAN; Corbetta et al., 2002; see Corbetta et al., 2008 for a review) and each node in this network is generally believed to be involved in specific aspects of top-down attention. For example, the IPS and FEF have been shown in multiple studies to be involved in maintaining attention to peripheral locations (Hopfinger et al., 2000; Corbetta et al., 2002; Kelley et al., 2008) and exerting top-down control on visual cortex (Kastner et al., 1999; Serences et al., 2004). In contrast, the SPL has been found to be active when subjects have to disengage their attention from fixation and move it to a cued location (Yantis et al., 2002; Liu et al., 2003; Serences et al., 2004; Shomstein and Yantis, 2004). Overall this network is involved in endogenous processes to accomplish specific goals including preparing to see a stimulus at a certain location (Shulman et al., 1999; Corbetta et al., 2000), attending to different features in a scene (Labar et al., 1999; Pessoa et al., 2002) and preparing to make a motor response (Rushworth et al., 2001).

Unlike the DAN, relatively less is known about the mechanism of exogenously driven attention, and the regions that are involved in its mediation. Some researchers (e.g., Corbetta et al., 2008; see Chica et al., 2013 for review) have proposed that bottom-up sensory-driven exogenous attention may be propagated via a ventral fronto-parietal network of brain regions in the right hemisphere, such as the temporo-parietal junction (TPJ) and ventral frontal cortex including the middle frontal gyrus (MFG), inferior frontal gyrus (IFG), frontal operculum and anterior insula. This network of regions is sometimes referred to as the Ventral Attention Network (VAN; Corbetta and Shulman, 2002; Corbetta et al., 2008: review) and each node in this network is believed to be involved in different aspects of bottom-up attention. For example, the TPJ is found to be active any time reorienting is necessary (independent of expectation; Kincade et al., 2005), while the right IFG and right MFG are found to be active only when reorienting to unexpected stimuli (Shulman et al., 2009; Doricchi et al., 2010). Overall, this network is involved in reorienting to relevant targets that occur at unexpected locations (Arrington et al., 2000; Macaluso et al., 2002) or to targets that are important but not very distinctive (Indovina and Macaluso, 2007).

These two systems work hand-in-hand to transition between goal-directed and stimulus-driven attention by attending to task-relevant targets and filtering out distracters (Rosen et al., 1999; Friedman-Hill et al., 2003; Hahn et al., 2006; Buschman and Miller, 2007; Weissman and Prado, 2012). The DAN is thought to control stimulus-response selection, while the VAN is thought to send reorienting signals to the dorsal network to interrupt ongoing processing and divert attention to an exogenous stimulus. Regions within the lateral prefrontal cortex in the VAN are proposed to be a site of convergence for the DAN and VAN, but it is unclear which region serves as the gatekeeper between these two modes of attention. For example, Asplund et al. (2010) and He et al. (2007) have proposed that the inferior frontal junction (IFJ) comprising posterior aspects of the inferior frontal sulcus (parts of Brodmann areas 9, 44, and 6), may be one node that interacts with both the VAN and DAN structures. Others (Fox et al., 2006) have proposed that the right MFG may be the node that links the ventral and dorsal networks by acting as a “circuit-breaker” (Corbetta et al., 2008 review), interrupting ongoing processes in the dorsal network, and reorienting a person's attention to a novel task-relevant external stimulus. Thus, under this proposal (Corbetta et al., 2008), the right MFG exerts control over both networks and would be responsible for the flexible modulation of endogenous and exogenous attention.

The goal of the present study was to test the hypothesis that the right MFG does indeed serve as the gateway between top-down and bottom-up control of attention. Therefore, to test the role of this region in reorienting of attention, we examined its contribution to both endogenous and exogenous attention by comparing performance on an orientation discrimination task of a patient with a right MFG resection and a group of healthy controls.

First, to probe the role of the right MFG in endogenous attention, we employed a widely used Posner-type cueing task (Posner, 1980; Posner et al., 1982), where a central cue predicted with high accuracy the location of a subsequent peri-threshold low contrast grating (Gabor patch). As this task involves top-down attentional cueing, based on previous work (Vossel et al., 2006; Mukai et al., 2011), we expected that the patient would be able to perform similar to healthy controls on validly cued trials relative to invalid trials and thus would not show a deficit in top-down orienting of attention.

Second, to probe the role of this region in exogenous attention, we employed non-predictive peripheral cues to capture attention to exogenously cued locations (similar to Carrasco et al., 2004; Mukai et al., 2011). By varying the time interval between the peripheral cue and the subsequent Gabor patch target stimulus, we created situations where processing of the subsequent stimulus was influenced by the preceding cue to varying degrees. Based on previous research (Muller and Rabbitt, 1989; Yantis and Jonides, 1990; Mayer et al., 2004), we expected that for short durations between cue and stimulus (typically around 100 ms), healthy controls would show a facilitatory effect of the cue, such that they would be faster and more accurate on validly cued trials than invalid ones. No such facilitation should be seen at longer durations separating cue from stimulus, since the transient effect of the exogenous cue would be extinguished over time (Posner et al., 1985; Mayer et al., 2004). Further, if the right MFG does play a role in exogenous reorienting, we hypothesized that our patient would have trouble orienting to the exogenous stimulus. Thus, unlike healthy controls, our patient would not show facilitatory enhancements in stimulus processing induced by the exogenous cue.

Lastly, to understand the neural correlates of exogenous reorienting, we used resting state functional magnetic resonance imaging (rsfMRI) to probe how the tumor and its resection in the patient may have disrupted the functional connectivity between brain regions that are normally tightly coupled to the right MFG in healthy controls. Resting state functional connectivity methods have been used widely in recent years, not just to define functional networks in healthy brains, but also to estimate the effects of neurological disorders such as stroke (Ding et al., 2014; Tsai et al., 2014; also see Carter et al., 2012 for review), multiple sclerosis (e.g., Richiardi et al., 2012; Janssen et al., 2013; see Sacco et al., 2013 for review) and Parkinson's Disease (e.g., Hacker et al., 2012; Tessitore et al., 2012; Baggio et al., 2014). Due to the wide applicability of this method, we used rsfMRI to ask the question: What happens to the functional connectivity patterns when a part of the brain is resected? To answer this question, we first identified in a group of healthy controls, regions that would normally be connected to the resected right MFG tissue. Then we sought to identify differences in connectivity patterns between the patient and controls for those regions normally connected to the right MFG. Any differences observed would be related to a change in functional connectivity in the patient's brain following surgery.

Overall, our study aimed to uncover the role of the right MFG in attentional reorienting and to examine the functional reorganization, if any, that may have occurred in the patient's brain after tissue removal.

## Methods

### Participants

#### Patient

The patient (GE) in this case study was a 31 year-old right-handed male who underwent a frontal-lobe tumor resection, with subsequent radiation and several rounds of chemotherapy, 4 years prior to arriving at the National Institutes of Health (NIH). He was admitted to the NIH under a natural history protocol, and was generally reported to be asymptomatic and high-functioning. Neuropsychological tests were performed on the patient to determine his cognitive and behavioral profile on different test batteries, as listed in Table 1.

**Table 1:** **Results from neuropsychological tests administered to the patient**.

**Test**	**Patient Score**	**Norm**	**SD**	**Notes**
**WECHSLER TEST OF ADULT READING**	115	100	15	
**WASI: WECHSLER ABBREVIATED SCALE OF INTELLIGENCE**				
Vocabulary	49	50	10	
Block design	40	50	10	
Similarities	49	50	10	
Matrix reasoning	52	50	10	
VIQ	98	100	10	
PIQ	93	100	10	
FSIQ	96	100	10	
**WMS-III: WECHSLER MEMORY SCALE—THIRD EDITION**				
Information and orientation	14 raw			
Digit span	11	10	3	
Local memory I recall total	9	10	3	
Local memory II recall total	9	10	3	
**HOPKINS VERBAL LEARNING TEST—REVISED**				
Trial 1 recall correct	7 raw			
Trial 2 recall correct	10 raw			
Trial 3 recall correct	9 raw			
Total recall	44	50	10	
Delayed recall	44	50	10	
Retention	47	50	10	
Recognition discrimination	44	50	10	
**BRIEF VISUOSPATIAL MEMORY TEST—REVISED**				
Trial 1 recall correct	40	50	10	
Trial 2 recall correct	41	50	10	
Trial 3 recall correct	31	50	10	
Total recall	36	50	10	
Delayed recall	49	50	10	
Retention	>16%			
Recognition hits	>16%			
Recognition false alarms	>16%			
Recognition discrimination	>16%			
Recognition bias	>16%			
**REY COMPLEX FIGURE**				
Copy	>16%			
Delayed recall	38%			
**BENTON VISUAL FORM DISCRIMINATION**				
Total score	30 (Within Normal Limits)			
**CONTROLLED ORAL WORD ASSOCIATION TEST**				
FAS	55	50	10	
Category fluency	32	50	10	
Boston naming test	33	50	10	
**GROOVED PEGBOARD**				
Dominant hand (right)	46	50	10	
Non-dominant hand (left)	38	50	10	
**TRAIL MAKING TEST**				
A	26	50	10	0 errors
B	36	50	10	0 errors
**SYMBOL DIGIT MODALITIES TEST**	0.8	0	1	
**CONNERS' CPT-II**				Higher score = more inattention
Omissions	44	50	10	
Commissions	41	50	10	
RT	60	50	10	
RT std. error	57	50	10	
**WCST (WISCONSIN CARD SORTING TEST)**				
Trials administered	128 raw			
Total errors	29	50	10	
Perseverative responses	31	50	10	
Perseverative errors	29	50	10	
Non-perseverative errors	31	50	10	
% conceptual-level responses	30	50	10	
Categories completed	6–10%			
Trials to complete 1st category	≤1%			
Failure to maintain set	>16%			
Learning to learn	>16%			
**STROOP COLOR AND WORD TEST**				
Word score	42	50	10	
Color score	41	50	10	
Color-word score	45	50	10	
**FrSBe (FRONTAL SYSTEMS SCALE OF BEHAVIOR) FAMILY-RATING**				
**FORM**				
After apathy	39	50	10	(Higher is worse)
After disinhibition	38	50	10	(Higher is worse)
After executive dysfunction	40	50	10	(Higher is worse)
After total	38	50	10	(Higher is worse)
**BECK DEPRESSION INVENTORY**	1 raw			(Minimal depression)
**BECK ANXIETY INVENTORY**	2 raw			(Minimal anxiety)
**MEDICAL SYMPTOM VALIDITY TEST**				
Immediate recognition	90 raw			
Delayed recognition	100 raw			
Consistency	90 raw			
Paired associate	100 raw			
Free recall	70 raw			
**BEHAVIORAL INATTENTION TEST**				
Conventional test	145 raw	146		
Line crossing	36	36		
Letter cancelation	39	39		
Star cancelation	54	54		
Figure and shape copying	4	4		
Line bisection	9	9		
Representational drawing	3	3		
Behavioral test	77 raw	81		
Picture scanning	5	9		
Telephone dialing	9	9		
Menu reading	9	9		
Article reading	9	9		
Telling and setting time	9	9		
Coin sorting	9	9		
Address and sentence	9	9		
copying				
Map navigation	9	9		
Card sorting	9	9		

**Figure 1:**
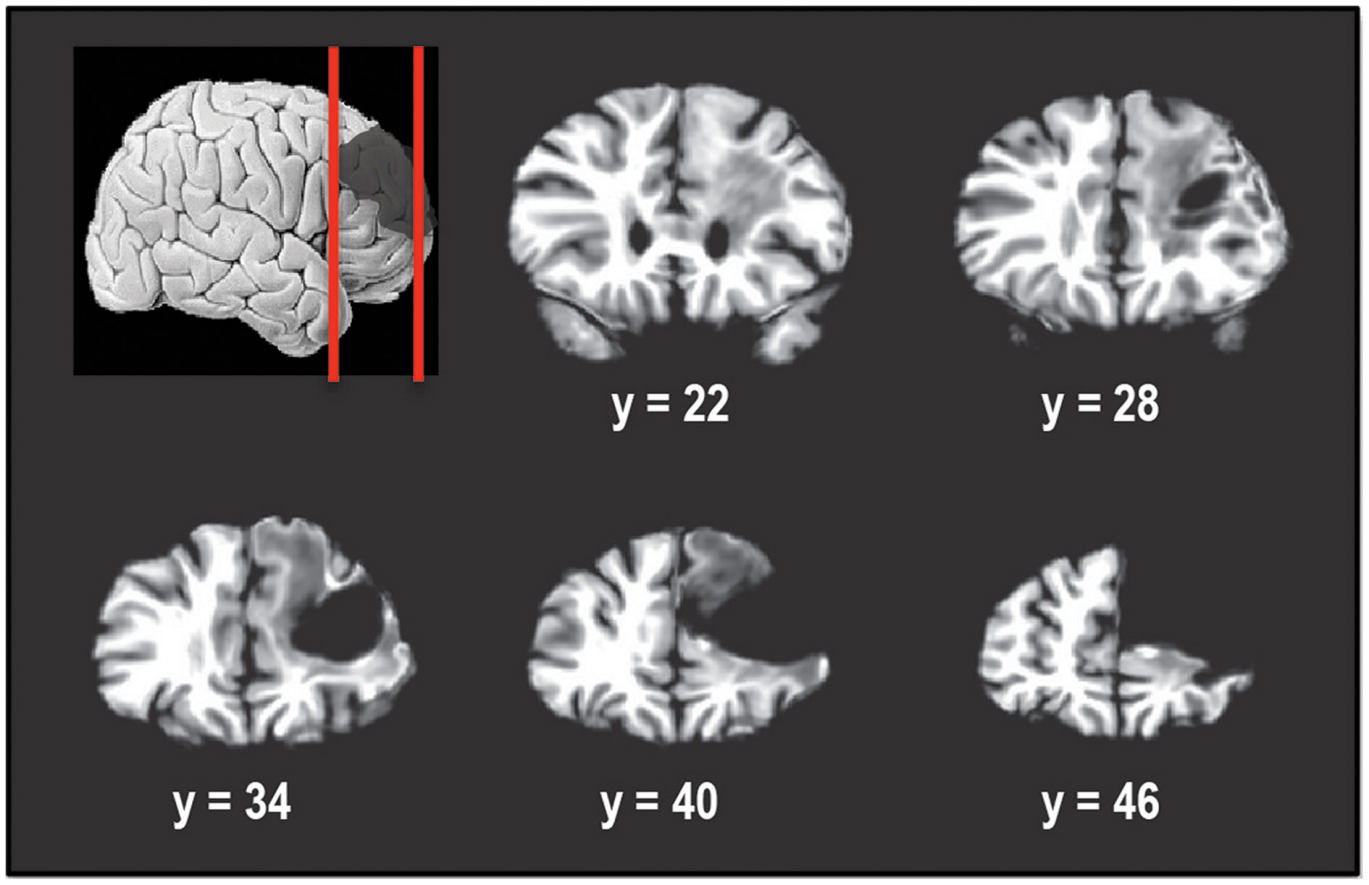
**Coronal slices taken 6 mm apart starting posteriorly at *y* = 22 and showing the extent of the tumor resection in the right hemisphere**. The resection encompassed right superior frontal gyrus and right middle frontal gyrus, including Brodmann areas 9, 46, and 10. Right inferior frontal gyrus was almost completely spared. In Talairach normalized space, the resection extended from x of 0 (midline) to about 50 mm, y of 23 to y of 66 mm (anterior tip of brain) and z of −4 to z of 50 mm (superior tip of brain).

Figure 1 shows the extent of the cortical resection in the patient's brain. In Talairach space, the resection spanned a region that extended from the midline of the right hemisphere (*x* = 0) to a lateral location of *x* = 50 mm. This included the right superior frontal gyrus (SFG), right middle frontal gyrus (MFG), while almost completely sparing the right inferior frontal gyrus (IFG). Caudally the resection extended from *y* = 23 mm to *y* = ~66 mm (anterior tip of the brain). Dorsally, the resection spanned the region between *z* = −4 mm and *z* = 50 mm (superior tip of the brain). The resection removed most of Brodmann areas (BA) 9, 46, as well as the dorsal portion of BA 10, in the right hemisphere.

#### Healthy controls

A total of 36 right-handed control subjects participated in this study. All were in good health with no past history of psychiatric or neurological disease and gave informed consent. Subjects had normal or corrected-to-normal vision. All experiments and procedures were approved by the National Institute of Mental Health (NIMH) Institutional Review Board.

***Attentional cueing experiments***. Ten normal volunteers (5 females) age-matched to the patient (31.8 ± 3.4; mean ± SD years) participated in the study. Subjects completed one of three attentional cueing experiments on three separate visits spanning approximately 1 week.

***Response inhibition experiment***. Six different normal volunteers (3 females) aged 23.8 ± 1.8 (mean ± SD) years participated in the study. One subject was excluded because she did not follow task instructions properly. The remaining five normal volunteers (2 females) were aged 23.6 ± 1.9 (mean ± SD) years.

***Resting state fMRI***. Resting state fMRI data were collected in a separate group of 20 normal volunteers (10 females) aged 23.1 ± 1.5 (mean ± SD) years. These controls were the same as those used in Barnes et al. (2014). Two runs of resting state data acquired in that study were used in the current analysis.

### Attentional cueing experiments

#### Adaptive thresholding

Before subjects were engaged in the attentional cueing tasks, we used a thresholding procedure to determine each subject's contrast threshold level (for 75% accuracy) for the Gabor patches to be used in the attention tasks. An adaptive threshold algorithm, known as the staircase method (e.g., Cornsweet, 1962; Gracely et al., 1988), was used to determine the optimum contrast level of the Gabor patches to ensure that each subject performed at an accuracy of approximately 75%. This thresholding task was performed at the start of each attentional cueing session. On each trial, a fixation spot was presented for 50 ms, followed by a 50-ms presentation of a single Gabor patch. The Gabor patch subtended 2° of visual angle, and was located 5.5° to the right or left of central fixation. Each Gabor patch was oriented either 45° or 135° from the vertical. The subject's task was to indicate with a button press whether the Gabor patch was rotated clockwise or counterclockwise relative to vertical. A color change of the central fixation spot signified the start of the response period, during which the participants pressed a button to indicate the orientation of the Gabor patch. During the threshold measurements, no attentional cues were presented at any location. The contrast of the Gabor patch was reduced 1% after a subject responded correctly three times in a row and was increased 1% when a subject responded incorrectly once, thus yielding an overall approximate accuracy of 75%. This threshold procedure was conducted separately for Gabor patch locations to the left and right of fixation. The larger of the two contrast threshold values (for left and right stimulus locations) was used for the attention experiment that followed.

#### Endogenous cueing task

**Figure 2:**
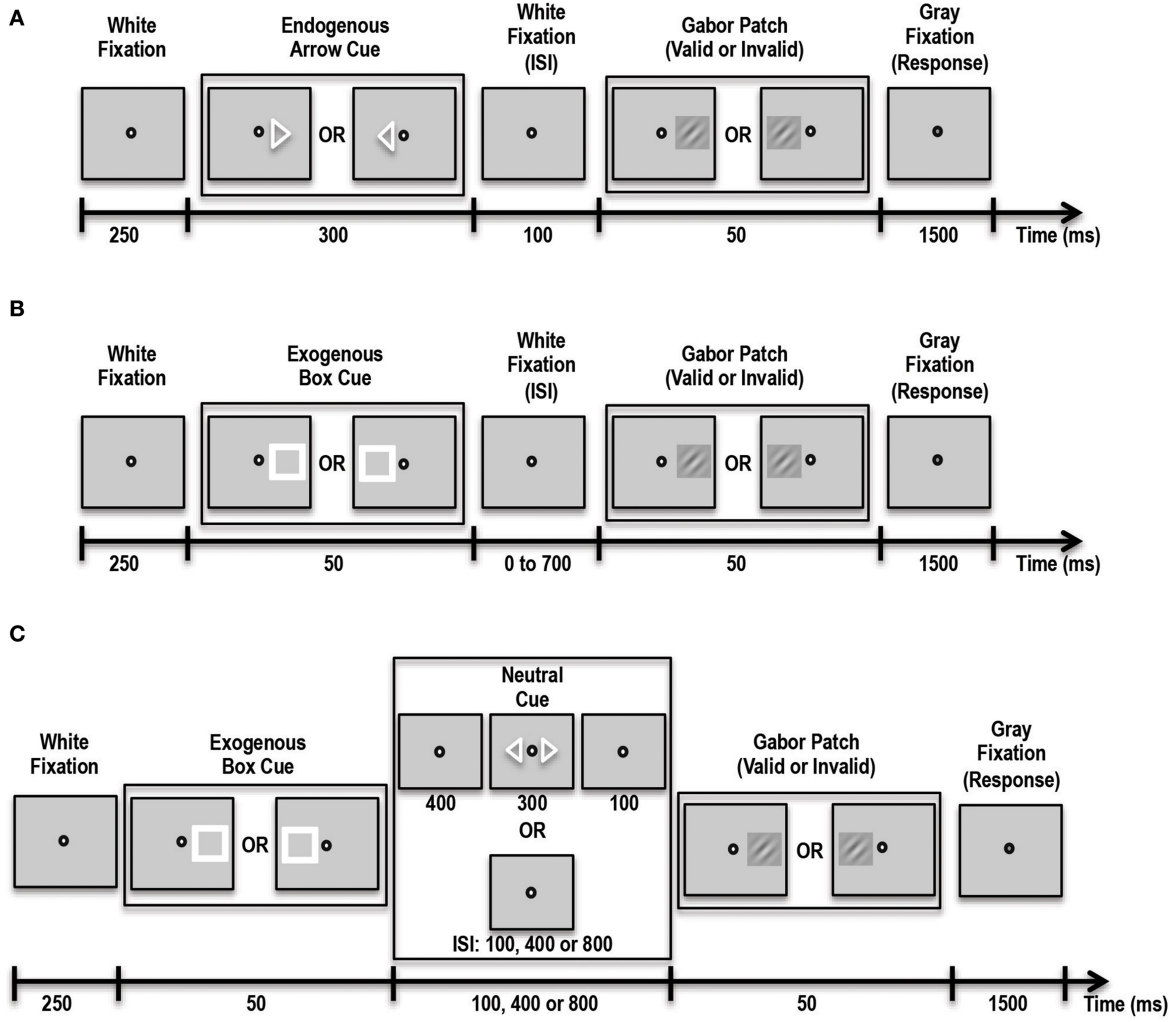
**Schematic showing the various tasks administered to patient and controls**. **(A)** Schematic of the Endogenous Cueing Task. **(B)** Schematic of the Exogenous Cueing Task. **(C)** Schematic of the Exogenous Cueing Task with Explicit Reorienting. ISI, Inter-stimulus Interval is the time between the cue and stimulus. ISI for the Endogenous Cueing Task was fixed at 100 ms; ISI for Exogenous Cueing Task was 0, 100, 250, 500 or 700 ms; ISI for the Exogenous Cueing Task with Explicit Reorienting was 100, 400 or 800 ms. Subjects were instructed to indicate the orientation of the Gabor Patch with a button press during the Response window (Gray Fixation).

In this experiment participants were asked to covertly direct their attention in a top-down manner to a cued location (see Figure 2A). Three different trial types were presented to subjects in blocks. On Predictive-Cue trials, participants were shown a central arrow cue that predicted with 90% validity the location of a subsequent peri-threshold Gabor patch. Trials began with a 250 ms white fixation spot at the center of the screen. This was followed by the arrow cue for 300 ms followed by another white fixation spot for 100 ms. This was then followed by the Gabor patch that appeared either on the left or right side (5.5° visual angle) of the fixation spot with 90% validity relative to the cue. Neutral-Cue trials were similar to Predictive-Cue trials, except that instead of a single predictive arrow cue, participants were shown two central arrows pointing to the left and right side of the fixation spot. During No-Cue control blocks, participants were shown a white fixation spot for 250 ms followed by a Gabor patch for 50 ms, followed by a gray fixation spot, indicating the response period for 1500 ms. Participants were instructed that a single arrow cue predicted with 90% accuracy the location of the subsequent grating patch while the double arrow cues were unrelated to it. On every trial the subject's task was to indicate with a button press whether the Gabor patch was rotated clockwise or counterclockwise relative to vertical. Subjects performed 3 runs of the task, with each run containing 10 blocks of Predictive-Cue trials, 1 block of Neutral-Cue trials, and 1 block of No-Cue control trials. Overall, subjects completed 540 validly cued trials, 60 invalidly cued trials, 60 neutral trials and 60 control trials. We expected that our patient would perform similar to controls on this task.

#### Exogenous cueing task

In this experiment participants were shown an exogenous stimulus (a white box) at one of two locations, to the left or right of fixation (see Figure 2B). This white box prior to the appearance of the Gabor patch served as the exogenous cue with 50% validity. The box was followed by a white fixation spot for a variable amount of time, i.e., inter-stimulus interval (ISI) of either 0, 50, 100, 250, 500 or 700 ms. This was then followed by the Gabor patch that appeared for 50 ms in the cued or non-cued location with 50% validity with respect to the cue. This was followed by a gray fixation spot that indicated the 1500-ms response period. Participants were instructed that the location of the white box cue was completely random and unrelated to the location of the subsequent grating patch. On every trial the subject's task was to indicate with a button press the orientation of the Gabor patch. Trials were blocked by ISI duration, and each block contained 12 trials. Participants performed 5 runs of the task, with each run containing 2 blocks of trials per each of the 6 ISI conditions and one control block. Overall subjects completed 60 validly cued trials and 60 invalid trials per ISI and 60 control trials. We expected that our patient would have trouble orienting to the exogenous stimulus. Thus, unlike healthy controls, our patient should not show facilitatory enhancements in stimulus processing induced by the exogenous cue at short ISIs.

#### Exogenous cueing task with explicit reorienting

In addition to difficulty orienting to an exogenous stimulus, it is also possible that, at longer durations between cue and stimulus, when the effect of the exogenous cue has extinguished, the patient may have trouble reverting back to top-down control of attention. To test this specifically, we had our participants complete another exogenous cueing experiment, which involved an explicit reorienting cue at longer durations to allow the patient to reorient to top-down control of attention (Natale et al., 2009). We hoped that by explicitly instructing the patient to reorient and divide his attention between the two possible stimulus locations, he would be able to perform better at the orientation discrimination task for these longer inter-cue-stimulus duration trials. This task consisted of three trial types including: implicit exogenous cueing, exogenous cueing with explicit reorienting, and control trials (see Figure 2C). Implicit Exogenous Cue trials were similar to the exogenous cue trials described above such that a aatch cue with 50% validity was first presented for 50 ms, followed by a white fixation spot for either 100, 400 or 800 ms ISI duration. This was followed by a 50-ms Gabor patch. During Explicit Reorienting trials, an exogenous box cue was presented for 50 ms, followed by a white fixation spot for 400 ms. Then, two central arrow cues were presented for 300 ms followed by a white fixation spot for 100 ms. Subsequently a Gabor patch was presented at either the left or right stimulus location for 50 ms. The central fixation spot then changed color to gray indicating the start of the 1500-ms response period. Subjects completed 5 runs of this task, each with 5 blocks and 12 trials per block. Overall subjects completed 60 valid trials and 60 invalid trials per ISI and 60 controls trials.

### Response inhibition and working memory—Go/No-Go task

In order to eliminate response inhibition and working memory deficits as a source of the difference between the patient and control group on our attention tasks we had participants complete a Go/No-Go task. In this task, patient and controls were asked to fixate on a blue or yellow box presented for 200 ms at the center of the screen that was followed by a gray fixation spot for 1300 ms. The blue or yellow box was preceded by a white fixation spot for 250 ms. The subjects' task was to press a button every time a blue box appeared, but withhold their response, i.e., not press a button, when a yellow box appeared. During runs of the simple version of the task, 82% of trials displayed a blue box and 18%, a yellow box. This distribution was used in order to get subjects accustomed to pressing a button, thus making it harder to inhibit responses on yellow box trials. During complex Go/No-Go runs (modeled after Mostofsky et al., 2003), we combined response inhibition with a working memory task. On such trials, subjects were asked to press a button when a blue box appeared and also press a button when a yellow box appeared that was preceded by an even number of blue boxes. In this way subjects had to continually count the number of blue boxes, which served as the working memory component. If an odd number of blue boxes preceded the yellow box, subjects were instructed to withhold their response. In this task too there were 82% blue: 18% yellow boxes. However, this time 9% of the yellow box trials were yellow Go trials (preceded by even number of blue boxes), and 9% of the trials were yellow No-Go trials (preceded by odd number of blue boxes). The number of blue boxes preceding a yellow box ranged from 3 to 6, thus yielding 2 even trial types (4, 6) and 2 odd trial types (3, 5). During Control runs, subjects were asked to press a button every time a box appeared on the screen whether it was blue or yellow. Each subject completed 1 run each of the simple task and control task and 2 runs of the complex task. The simple Go/No-Go and control task runs each consisted of 10 blocks with 25 trials per block. Each complex Go/No-Go task run consisted of 12 blocks with the number of trials ranging from 21 to 28 per block, for a total of 286 trials per run.

We expected that, if the patient had any response inhibition or working memory deficits, he would do poorly on the simple Go/No-Go and complex Go/No-Go tasks, respectively.

### Apparatus

Stimuli were presented on a 24″ liquid crystal display monitor, with a resolution of 1024 × 768 pixels and a refresh rate of 60 Hz, in a dimly lit room. A chin rest was used to maintain position of the participant's eyes 57 cm from the screen and to minimize head motion. Stimuli for the adaptive thresholding and Endogenous Cueing experiment were presented using MATLAB 7.4 (Mathworks, Natick, MA) and Psychophysics Toolbox (Brainard, 1997; Pelli, 1997). Stimuli for Exogenous Cueing and Go/No-Go experiments were presented using Presentation (Neurobehavioral Systems, Berkeley, CA). Sustained fixation at the center of the screen was monitored using the ASL Eyetrak 6000 (Applied Science Laboratories, Bedford, MA) for 7 out of the 10 subjects for Attentional Cueing experiments. Eye tracking was not performed for the Response Inhibition experiment, since stimuli in those tasks were presented at the center of the screen.

### Behavioral data analysis

For Attentional Cueing tasks, behavioral data from trials where subjects pressed a button during the response window were analyzed to obtain accuracy and average reaction times (RT based on correct responses only). First, we compared accuracy and RT data for valid vs. invalid trials within the control group using a paired *t*-test. Since our primary goal was to determine if the facilitation effects of attentional cues were similar for the patient relative to controls, we used a statistical approach frequently used in case studies as outlined by Crawford and Garthwaite (2007). In short, this method can be used to compare performance on two tasks between a single patient and a set of controls by using the mean and standard deviation of the group along with the correlation in the control group between the two measures being compared (Bayesian Standardized Difference Test). Further, this method can be used to compare a single patient's data directly to a group of controls (Single Bayesian Point and Interval Estimate method). For each task, depending on the number of tests being performed, we used a Bonferroni correction to determine whether a test resulted in a significant difference between the patient and control group. All tests were considered significant if the associated *p*-value was less than the Bonferroni corrected *p*-value.

### Eye-tracking data analysis

We confirmed that subjects fixated at the center of the screen while doing each of the three Attentional Cueing experiments by analyzing the eye-tracker data using ASL Results Plus™. Data from 4 sessions were excluded from analysis due to poor data quality. The remaining data (6 subjects for Endogenous Cueing, 5 for Exogenous Cueing, and 5 for Exogenous Cueing with Explicit Reorienting) were used to determine the percentage of fixation duration within a rectangular Area of Interest (AOI) of ±3° of visual angle around the fixation spot (Note the Gabor patch was presented at ±5.5°). The patient's eye tracking data were analyzed similarly and then compared to the average of the control group using the Single Bayesian Test.

### Resting state fMRI

#### Acquisition

Structural and resting state functional MRI (rsfMRI) data were acquired on a GE MR 750 3-Tesla scanner using a GE 32 channel head coil. A high-resolution structural image was acquired using a magnetization-prepared rapid-gradient-echo (MP-RAGE) T1-weighted sequence with the following parameters: time to echo [TE] = 3.42 ms; time to repetition [TR] = 7 ms; time to inversion [TI] = 425 ms; flip angle = 7°; Phase Acceleration Factor = 2; slices with 1 × 1 × 1 mm voxels). rsfMRI data were obtained using an axial echo-planar imaging (EPI) sequence (*TE* = 28.1 ms; *TR* = 2500 ms; flip angle = 77°; Phase Acceleration Factor = 2; 44 slices, each 3 mm thick, with 2 × 2 mm in-plane voxel resolution). Two runs with 134 volumes for each run were acquired for each participant (Data for the control group were acquired as part of a larger project that is outlined in Barnes et al., 2014). Physiological variables relating to heart rate and respiration were recorded during scans using a pulse oximeter placed on the left index finger and a pneumatic belt positioned at the level of the diaphragm.

#### rsfMRI processing

Each subject's rsfMRI data were analyzed using multiple programs in AFNI (Cox, 1996). Preprocessing of rsfMRI data was performed using methods similar to those previously reported (Jo et al., 2010; Gotts et al., 2013; Barnes et al., 2014). In short, rsfMRI data were first despiked and corrected for physiological motion effects (Glover et al., 2000). The first four volumes of each run were then discarded, and the remaining volumes were slice-timing corrected and all volumes were registered to the first volume of the first run. Anatomical data were first aligned to the EPI data and then FreeSurfer (Fischl et al., 2002) was used to segment the MP-RAGE volumes into subject-specific white matter and ventricle masks. For each run and for each voxel, the following nuisance regressors and their derivatives were created: an average ventricle time series, a local average white-matter time series, 6 parameter estimates for head motion, 8 regressors for respiration and heart cycle using RETROICOR (Glover et al., 2000; Jones et al., 2008) and 5 regressors relating to respiration volume per time (RVT) that model slow BOLD oxygenation level fluctuations (Birn et al., 2008; Chang and Glover, 2009). These nuisance signals were detrended prior to regression using a 4th order baseline detrending model. The combined, predicted time series of these nuisance variables was then subtracted from the voxel-wise time series, yielding a residual time series. This residual time series for each run was then blurred with a 6 mm FWHM Gaussian kernel, rescaled to reflect percent signal change, and the two runs for each subject were concatenated. Finally, anatomical and rsfMRI data for each control subject and patient were warped into standard space (Talairach template TT_MB101; Klein and Tourville, 2012) using a non-linear registration process in AFNI (Cox, 1996).

#### rsfMRI analysis

Whole brain correlation analyses were performed for each subject by first extracting a time series from a seed location and then correlating data from all voxels in the brain with this seed time series. The resulting correlation maps and associated statistics were displayed on an inflated representation of a standard brain using AFNI and SUMA (Saad et al., 2004).

***Determining functional connectivity with right MFG resected region***. Given that the patient had a resection in parts of the superior frontal and middle frontal gyri, we first picked a coordinate location within this region as our seed coordinate. This seed was located in right MFG at a voxel with Talairach coordinates: *x* = 31, *y* = 45, *z* = 26. For each control subject, we then extracted an average time series from within a sphere of radius 5 mm placed at this seed location. This time series was used to perform a whole brain correlation to obtain a measure of the connectivity between each voxel in the brain and the seed voxel. The resulting correlation coefficient values were converted to z-normalized correlation values using the Fisher transform. Finally, these normalized correlation values from all controls were entered into a second level analysis to perform a *t*-test to identify regions in the brain that were significantly correlated with resting state activity in the seed location in right MFG. Only clusters that exceeded a cluster size of 17 voxels and an individual voxel threshold of *p* = 0.00001 (corresponding to a *t*-value of 5.95 and resulting in a multiple comparisons cluster based correction of alpha = 0.01) were considered to be significantly correlated with activity in the seed location.

***Determining functional connectivity with regions highly connected to right MFG***. Next we used four regions that showed strong correlations with the right MFG seed and determined correlation maps for the patient and control group for these new seed locations. These included a seed voxel in the left MFG (Talairach coordinates of peak location: *x* = −35, *y* = 41, *z* = 32; average z-normalized correlation for controls of 0.62), right anterior cingulate cortex (ACC; Talairach coordinates of peak location: *x* = 7, *y* = 29, *z* = 28; average z-normalized correlation of 0.53), right superior parietal lobule (SPL; Talairach coordinates of peak location: *x* = 29, *y* = −61, *z* = 48; average z-normalized correlation of 0.27) and right supramarginal gyrus (SMG; Talairach coordinates of peak location: *x* = 55, *y* = −47, *z* = 40; average z-normalized correlation of 0.42). To rule out any global differences in the patient vs. controls, we also placed a seed voxel in the left motor cortex at: *x* = −39, *y* = −18, *z* = 49. This seed served as a control condition for the correlation analyses.

***Comparing patient to control group***. Finally, in order to determine how the whole brain correlations of patient compared to whole brain correlations of controls, we computed the mean and standard deviation of the correlations in the control group and identified voxels where the patient's correlation values lay outside the 4 standard deviation (SD) range of the controls. We chose this criterion of 4-SD to minimize the probability of a voxel-wise false negative, i.e., the chance of finding a difference at 4-SD between the patient and control group at any given voxel by chance alone would be about 0.00006. In contrast, the chance of a false negative result at 3-SD is higher at about 0.003. Further, in order to determine whether the clusters seen in such a comparison could have occurred by chance, we repeated the above process for each control subject relative to the other 19. For each subject, we then determined the sizes of voxel clusters where correlation values lay beyond the 4-SD range from the mean of the remaining subjects. By systematically leaving out one subject and comparing to the remaining subjects, we created a distribution of cluster sizes that met the 4-SD criterion. From this simulation we determined how big a cluster should be in order to be associated with a probability of occurrence of less that 5%. The cluster threshold for an alpha <0.05 was 86 voxels. Only those clusters that were larger than this critical value of 86 voxels were considered to be significantly different between the patient and controls for the 4-SD outlier comparisons.

## Results

### Neuropsychological testing

Details of these measures are presented in Table 1. In short, the patient had a normal IQ and was normal in many regards. For example, he showed some specific deficits, such as psychomotor slowing (as shown by the Stroop Task, and the Trail-Making Task), executive functioning (as shown by the Trail-Making Task as well as the Wisconsin Card Sort Task), and some aspects of memory (as shown by the Visuospatial Memory Test). But, notably, he scored normally on the Behavioral Inattention Test (Wilson et al., 1987), showing no evidence of spatial neglect. Overall his attention profile as measured by the neuropsychological tests, indicated that the patient was fairly normal, with some slowing in reaction times and perhaps some mild inattention, as measured by the Continuous Performance Test.

### Eye-tracking

Overall, control subjects fixated at the central fixation spot for 95.0% ± 7.1 (mean ± SD) of the total time for the Endogenous Cueing task, 97.1% ± 3.4 (mean ± SD) for the Exogenous Cueing task, and 96.3% ± 1.1 (mean ± SD) for the Exogenous task with Explicit Reorienting. The patient's mean total fixation durations were as follows: 90.6% for the Endogenous Cueing task, 94.8% for the Exogenous Cueing task, and 97.2% for the Exogenous task with Explicit Reorienting. Thus, the patient did not differ from controls in how well he fixated the center of the screen, for any of the three tasks (*p* > 0.15).

### Endogenous cueing task

**Figure 3:**
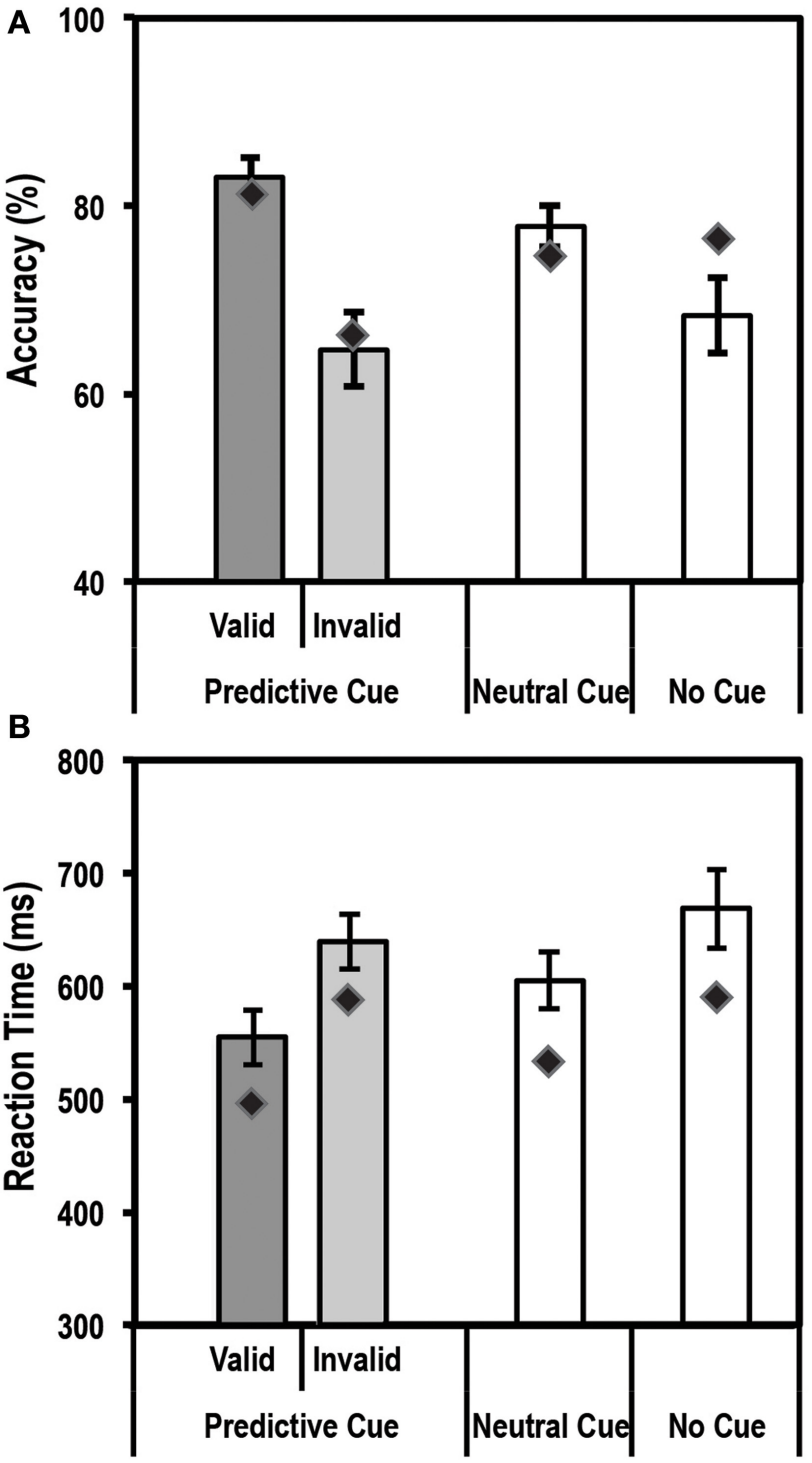
**Average accuracy (A) and reaction time (RT) data (B) for age-matched controls (*N* = 10) and the patient for the Endogenous Cueing Task**. Dark gray bars represent performance on validly cued trials. Light gray bars represent performance on invalidly cued trials. Unshaded bars show performance on Neutral and No-Cue trials. Error bars represent standard error of the mean for the control group. Diamond markers indicate performance of the patient. The patient and control group showed similar facilitation effects in accuracy and RT (i.e., faster and more accurate on valid relative to invalid trials).

An endogenous cue is expected to reduce reaction times and increase accuracy for stimuli appearing at cued locations relative to uncued locations. This is often referred to as the facilitation effect of top-down attention. Average accuracy and reaction times for this task for the control group and patient are shown in Figures 3A,B.

#### Accuracy

We first tested the accuracy of the control group and found the expected facilitation effect, i.e., subjects were significantly more accurate [*t*_(9)_ = 4.3; *p* < 0.00098] on validly cued trials (83.1% ± 6.7; mean ± SD) than invalid trials (64.7% ± 12.3; mean ± SD). Further, using the Differential Bayes statistical method of comparing a single patient to a group of controls, we found that the patient did not differ from controls in the size of this facilitation effect (Accuracy on valid trials = 81.3%; accuracy on invalid trials = 66.7%; *p* = 0.75).

#### RT

Analysis of Reaction time (RT) data showed that the expected facilitation was seen for the control group for the informational (valid) cue condition such that subjects were significantly faster to respond on validly cued trials than invalid ones [RT difference of 84.5 ± 35.5 ms; *t*_(9)_ = −7.5; *p* < 0.000018]. Similar to accuracy, the patient did not differ in the size of the RT facilitation effect (RT difference of 91.4 ms) when compared to the controls.

Thus, overall for the endogenously cued task, the patient showed accuracy and RT profiles similar to the group of age-matched controls.

### Exogenous cueing task

**Figure 4:**
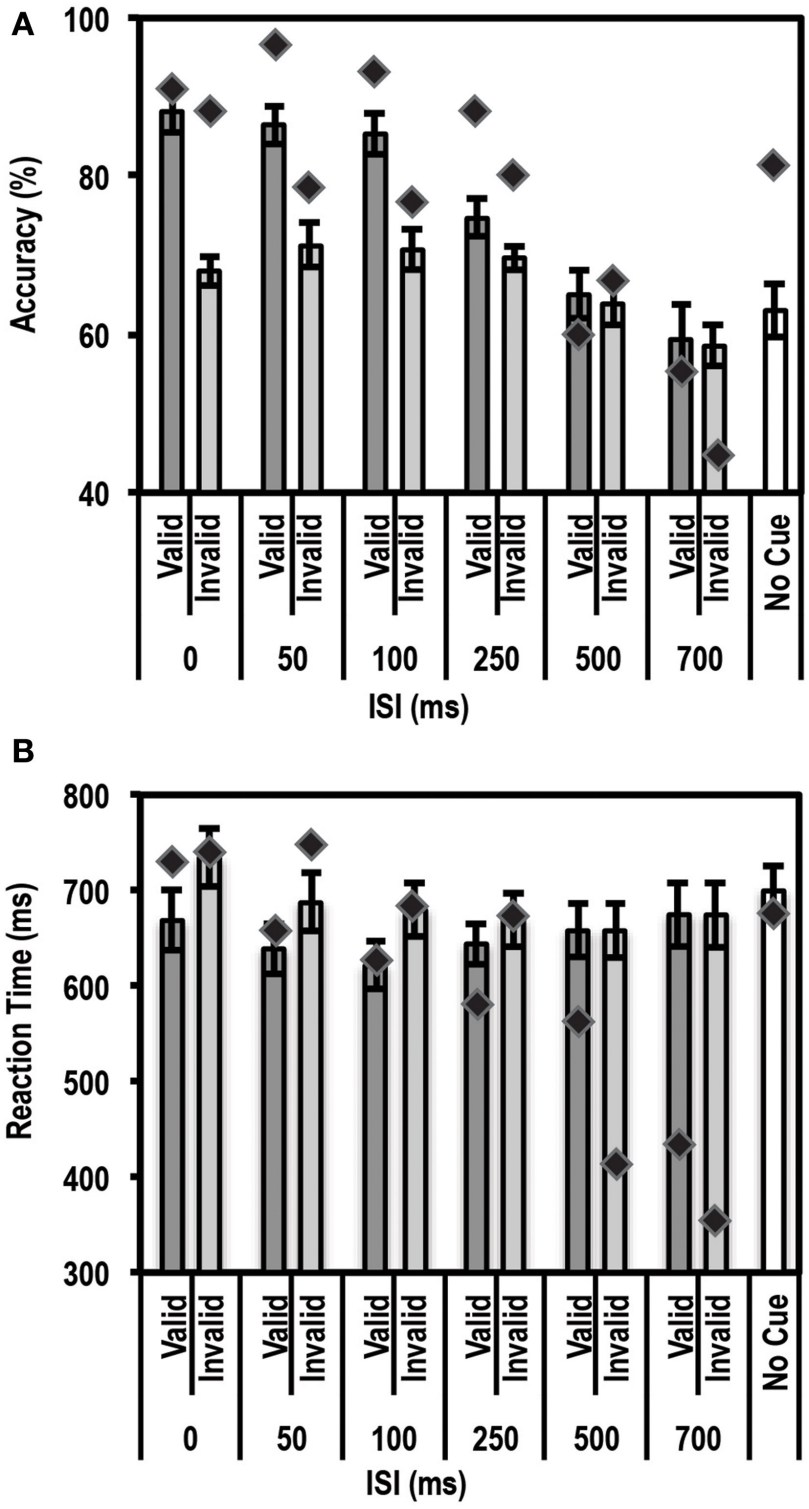
**Average accuracy (A) and Reaction Time (RT) data (B) for age-matched controls (*N* = 10) and the patient for the Exogenous Cueing Task**. Dark gray bars represent performance on validly cued trials. Light gray bars represent performance on invalidly cue trials. Unshaded bars show performance on No-Cue trials. Error bars represent standard error of the mean for the control group. Diamond markers indicate performance of the patient. The patient showed similar facilitation effects as the control group for short ISI but not for long ISI trials.

Similar to an endogenous cue, an exogenous cue is expected to reduce reaction times and increase accuracy for the cued, valid location relative to the uncued, invalid location. This facilitation, however, is supposed to last for a finite period of time (typically 200–300 ms) following the cue. Thus, we anticipated that subjects would show a facilitation effect at the short ISI durations but not at the long ISI durations. Average accuracy and reaction times for this task for the control group and patient are shown in Figures 4A,B.

#### Accuracy

As expected, we found a strong enhancement in accuracy at short ISI durations. A paired *t*-test between accuracy on valid trials and accuracy on invalid trials showed that participants in the control group performed better on valid than invalid trials at the shorter ISIs, i.e., 0 ms [accuracy difference = 20.2% ± 8.62; *t*_(9)_ = 7.40; *p* = 0.00002], 50 ms [accuracy difference = 15.3% ± 12.2; *t*_(9)_ = 3.96; *p* = 0.0017], and 100 ms [accuracy difference = 14.7% ± 7.0; *t*_(9)_ = 6.6; *p* = 0.000049]. The accuracy differences for all three of these short ISIs passed the Bonferroni corrected threshold of *p* < 0.0083 (correction based on performing paired *t*-tests for 6 ISIs). This facilitation effect was not seen at the longer ISI durations of 250, 500, and 700 ms.

Next we used the Bayesian Standardized Different Test to assess whether the patient showed a similar cue-induced performance enhancement as the controls. Here the patient showed no difference compared to controls for all ISIs. There was a trend for the facilitation to be larger for controls than the patient at 0 ms ISI (*p* = 0.036), but this trend did not pass the Bonferroni corrected threshold of *p* < 0.0125 (correction based on performing 4 Bayesian Difference Tests for 4 shorter ISIs where controls showed a facilitation effect induced by cue validity).

Since the control group, as expected, did not show a facilitation effect at longer ISIs, we compared the average accuracy for valid and invalid trials for controls relative to the patient using the Single Bayesian Test. Here we found that the patient did not differ from controls in accuracy for both 500 ms (*p* = 0.84) and 700 ms ISI (*p* = 0.58). Finally, there was no difference in accuracy on the control task (trials where stimuli were not preceded by a cue) for the patient relative to the control group (*p* = 0.14).

#### RT

Similar to the accuracy data, we found the expected facilitation effect in RT data as well. A paired *t*-test between RTs on valid trials and invalid trials showed that participants in the control group were faster to respond on valid trials than invalid trials for shorter ISIs, i.e., 0 ms [RT difference = 65.8 ± 37.6 ms; *t*_(9)_ = 5.5; *p* = 0.000018], 50 ms [RT difference = 48.3 ± 48.5 ms; *t*_(9)_ = 3.2; *p* = 0.0059], 100 ms [RT difference = 58.3 ± 53.8 ms; *t*_(9)_ = 3.4; *p* = 0.0038] and 250 ms [RT difference = 25.9 ± 27.9 ms; *t*_(9)_ = 2.9; *p* = 0.0083]. The RT differences for all four of these short ISIs passed the Bonferroni corrected threshold of *p* < 0.0083 (correction based on performing paired *t*-tests for 6 ISIs). This facilitation effect was not evident at longer ISIs, i.e., 500 ms [RT difference = 0.01 ± 24.5 ms; *t*_(9)_ = 0.002; *p* = 0.499] and 700 ms [RT difference = −0.21 ± 37.12 ms; *t*_(9)_ = −0.018; *p* = 0.49].

We then compared the patient's RT data to the mean of the control group using the Bayesian Standardized Difference Test. The patient showed the same RT facilitation as the control group for all the shorter ISIs, i.e., 0, 50, 100, and 250 ms. There was a slight trend for the RT facilitation to be larger for the patient than controls at 250 ms (*p* = 0.038) but this did not pass the Bonferroni corrected threshold of *p* < 0.0125 (correction based on performing 4 Bayesian Difference Tests for 4 shorter ISIs where controls showed a facilitation effect induced by cue validity).

Since, as expected, the control group did not show a facilitation effect at longer ISIs, we compared the average RT on valid and invalid trials for controls relative to the patient using the Single Bayesian Test. Here we found that, for 700 ms ISI, the patient showed faster RTs compared to controls (*p* = 0.01). At 500 ms there was no difference in RT between the patient and control group (*p* = 0.16). Finally, there was no difference in RT on the control task (trials where stimuli were not preceded by a cue) for the patient relative to controls (*p* = 0.83).

These results indicated that the patient's accuracy profile was very similar to that of controls, but that the patient's RT profile differed from controls at longer ISIs. A closer look at the patient's RT data revealed that the patient often initiated a button response prior to presentation of the Gabor patch. To rule out response inhibition deficits as a reason for the patient initiating responses earlier than instructed, we tested the patient's behavior on a Go/No-Go task and compared his data to a group of 5 controls.

### Response inhibition and working memory—Go/No-Go task

Since the patient showed some evidence that he was unable to wait for the Gabor patch to initiate the response at longer ISIs in the exogenous cueing task, we tested the patient and a separate group of controls on a Go/No-Go task.

#### Accuracy

**Figure 5:**
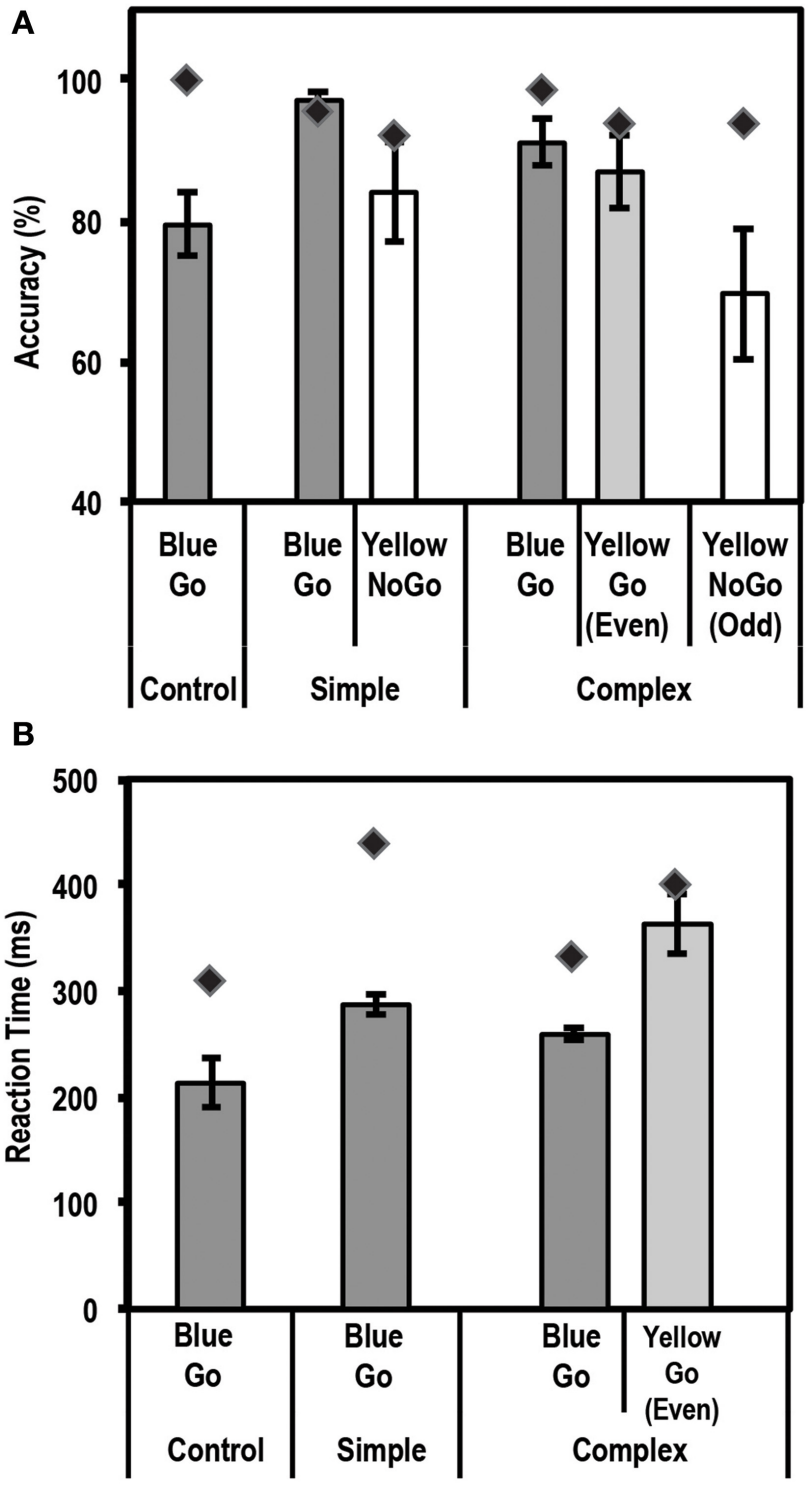
**Average accuracy (A) and reaction time (RT) data (B) for controls (*N* = 5) and the patient for the Go/No-Go Task**. Dark gray bars represent performance on Go trials. Unshaded bars represent performance on No-Go trials when subjects were expected to withhold their response (i.e., yellow box in the simple task, and yellow box preceded by an odd number of blue boxes in the complex task). Light gray bars represent performance on complex No-Go trials when subjects were expected to press a button (yellow box preceded by even number of blue boxes). Error bars represent standard error of the mean for the control group. Diamond markers indicate performance of the patient. The patient performed as accurately as controls on all trial types but was slower to respond on Simple Go and Complex Go trials.

Figure 5A shows the mean accuracy data for this task for controls and the patient. Using the Single Bayes statistical method, we found that the patient performed as accurately as controls on all conditions [Bonferroni corrected threshold of *p* = 0.0083 (based on 6 statistical tests)].

#### RT

Figure 5B shows the mean RT data for this task for controls and the patient. Using the Single Bayes statistical method, we found that the patient differed from controls, i.e., was slower to respond, on Simple Go and Complex Go trials (*p* = 0.0033 and *p* = 0.0044, respectively). None of the other comparisons showed any significant differences at the Bonferroni corrected level of *p* = 0.0125 (4 statistical tests).

#### Inverse efficiency

Since the patient was slower to respond on Simple Go and Complex Go trials, we next examined if there was a speed-accuracy trade-off in the patient's data. For this, we computed an Inverse Efficiency value of RT/Accuracy, such that a higher value for this index would be associated with lower efficiency. For this index, using the Single Bayes statistical test, we found that the patient was similar to controls on all conditions except the Simple Go condition (*p* = 0.0055), where the patient was less efficient than controls (RT/Accuracy = 572.9 for the patient vs. 370.2 ± 33.9 (mean ± SD) for controls). None of the other comparisons showed any significant differences at a Bonferroni correction level of *p* = 0.0125 (4 statistical tests).

Taken together, these results from the Go/No-Go task indicate that the patient did not have any significant response inhibition or working memory deficits.

### Exogenous cueing task with explicit reorienting

Results from the exogenous cueing task indicated that our patient had some trouble withholding responses during long ISI trials. However, since, the patient did not show response inhibition deficits on the Go/No-Go task, we hypothesized that at longer durations between the cue and target stimulus, when the effect of the exogenous cue had extinguished, the patient may have had trouble reverting to top-down control of attention. To test this specifically, on longer ISI trials, we explicitly instructed the patient to reorient and divide his attention between the two possible stimulus locations. We expected that he would now be able to perform better at the orientation discrimination task for these longer ISI duration trials.

#### Accuracy

**Figure 6:**
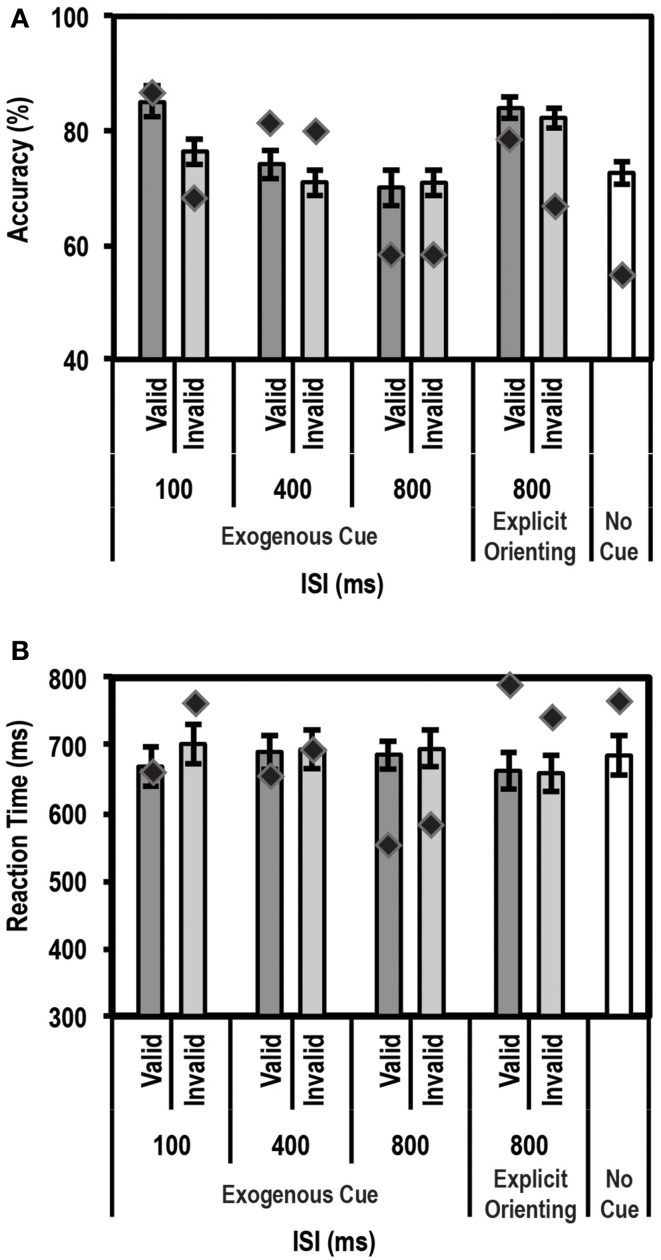
**Average accuracy (A) and reaction time (RT) data (B) for age-matched controls (*N* = 10) and the patient for the Exogenous Cueing Task with Explicit Reorienting**. Dark gray bars represent performance on validly cued trials. Light gray bars represent performance on invalidly cued trials. Unshaded bar shows performance on No-Cue trials. Errors represent standard error of the mean for the control group. Diamond markers indicate performance of the patient. The patient was as accurate as controls on all trial types but showed faster RTs for the implicitly cued 800 ms ISI condition. When explicitly cued during the long 800 ms ISI, the patient's RT data did not differ from controls.

Figure 6A shows the mean accuracy data for this task for controls and the patient. As expected, the control group showed a strong enhancement in performance (accuracy on valid trials was greater than accuracy on invalid trials) at the shortest ISI duration of 100 ms [accuracy difference = 9.0% ± 6.3; *t*_(9)_ = 4.5; *p* = 0.0008]. Also, as expected, no such facilitation was seen for the 400 ms (*p* = 0.13) and 800 ms (*p* = 0.41) ISI conditions. We then compared the average accuracy on implicitly cued 800 ms ISI trials with explicitly cued ones. Here, the control group showed a small increase in accuracy between the 800 ms ISI implicit and explicit trials [accuracy difference = 10.7% ± 8.0; *t*_(9)_ = 3.9; *p* = 0.0018]. Critically, however, the patient did not differ from controls on any of the trial types, indicating that, as far as accuracy was concerned, his behavioral profile on this task was normal.

#### RT

For RT data (see Figure 6B), again as expected, controls showed a strong facilitation effect for the short 100 ms ISI trials [*t*_(9)_ = 3.22; *p* = 0.0053]. No such effect was seen for the 400 ms, and either of the 800 ms ISI conditions (implicit and explicitly cued). The patient's behavior was similar to controls at 100 ms and 400 ms ISIs. However, as predicted, at the 800 ms ISI, the patient showed significantly faster average RTs compared to the control group for the implicitly cued condition (*p* = 0.003; average *RT* = 566.0 ms for the patient vs. 671.4 ms ± 107.9 (mean ± SD) for controls). This is a replication of the 700 ms ISI data from the Exogenous Cueing Task, that is, after the effect of the exogenous cue has extinguished, the patient was unable to revert to top-down attentional control and wait for the Gabor patch before initiating his response. Critically, however, when an explicit double arrow cue was presented during the long 800 ms ISI, the patient's RTs did not differ from the control group (*p* = 0.28). Further, a direct comparison of RT difference on the two types of 800 ms ISI trials, using the Single Bayesian Test, showed that the patient's RTs increased (RT difference of 198.1 ms) significantly more (*p* = 0.008) compared to controls (average RT difference of −13.7 ms ± 59.1). Thus, by introducing an explicit cue to enable reorienting to an endogenous mode of attention, the patient's behavior returned to normal.

### Resting state fMRI

To understand what may be occurring in the tumor-resected brain of the patient, we turned to resting state fMRI data to probe what effects tumor growth and the resection might have had on the functional connectivity of the brain.

#### Connectivity with right MFG seed in controls

Whole brain correlation analyses of the right MFG seed activity showed several frontal, parietal and subcortical regions that were strongly functionally connected to the seed (see Supplementary Figure 1 for associated group statistical map displayed on a standard inflated brain using SUMA). In the frontal lobe the most significant clusters occurred in the right MFG (correlation with voxels in and around the seed itself), left MFG, bilateral anterior cingulate cortex (ACC), right superior frontal gyrus (SFG), bilateral insula and bilateral inferior frontal gyrus (IFG). In the parietal lobe, significant clusters were found in bilateral posterior cingulate cortex (PCC), right superior parietal lobule (SPL), right supramarginal gyrus (SMG) and right precuneus. Subcortically, clusters in the right thalamus and bilateral caudate showed significant correlations with activity in the right MFG. Activity in all these regions showed a significant positive correlation with activity in the right MFG. No clusters showed significant negative correlation with seed activity. Of the highly correlated regions, we chose the left MFG and right ACC as additional seeds since they were the most significantly correlated regions with right MFG. In addition, since we were interested in how connectivity in the brain relates to attentional circuits, we picked two parietal regions, one in a right SPL and another in a right SMG cluster, as seeds for the next set of correlations in the patient and controls.

#### Comparison of connectivity with left MFG seed between controls and patient

The connectivity analysis for the left MFG seed showed that in controls, activity in this region was coupled with, among other regions, the right MFG, bilateral IPS, bilateral IFG, bilateral insula, bilateral fusiform gyrus, bilateral caudate and bilateral thalamus (see Supplementary Figure 2A for group statistical map of these correlations shown on an inflated brain using SUMA). For the patient, regions similar to those in controls were found to show significant correlations with the left MFG seed activity (see Supplementary Figure 2B).

**Figure 7:**
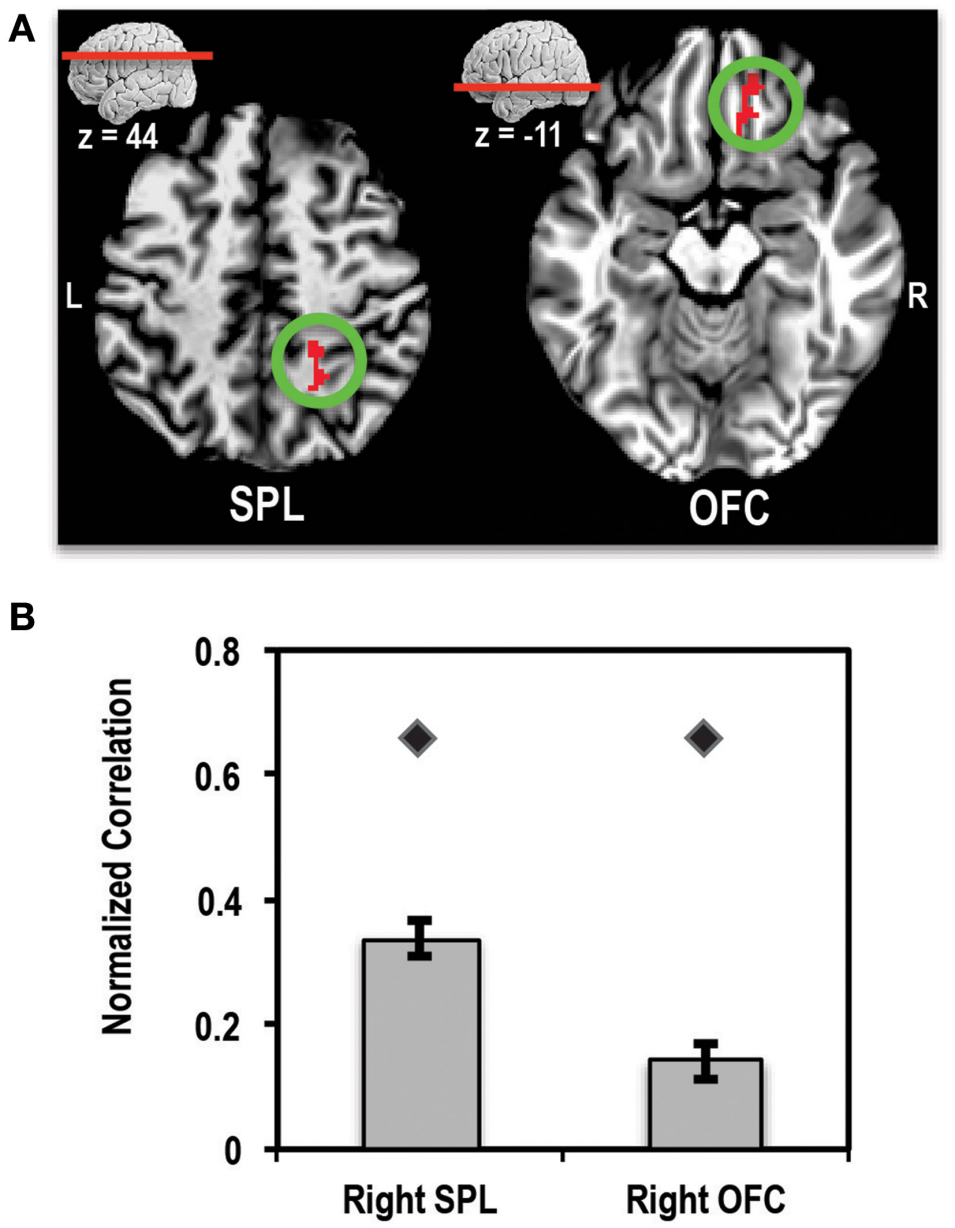
**(A)** Axial slices showing the location of clusters in right superior parietal lobule (SPL) and right orbitofrontal cortex (OFC) where the patient showed significantly higher connectivity (>4-SD beyond mean of control group) with left MFG seed than controls. L, Left; R, Right. **(B)** Bar plot showing the mean normalized correlation values in right SPL and right OFC regions of interest (ROIs) in controls (bars) and the patient (diamond markers). Error bars represent standard error of the mean of correlation values for the control group.

A direct comparison of the patient relative to controls showed two clusters that survived multiple comparisons correction (Figures 7A,B). One cluster of 128 voxels was found in right orbitofrontal cortex (OFC), running along the gyrus rectus. A second cluster of 117 voxels was found in the right SPL region of the parietal lobe. In order to ensure that these clusters did not arise simply due to differences in alignment between the patient and controls, we used the patient's correlation map to define ROIs in these two regions. ROIs were defined by finding the peak of the OFC and SPL clusters in the correlation map and placing a 4-mm sphere at that location (right OFC peak at *x* = 21, *y* = 39, *z* = −6) and right SPL peak at *x* = 31, *y* = −47, *z* = 48). We then extracted the mean normalized correlation values within these ROIs for the patient and control group. This ROI analysis showed that the patient had significantly higher mean correlation values compared to controls for both regions of interest. These results are shown in Figure 7B.

#### Comparison of connectivity with other seed regions between controls and patient

Similar correlation analyses with a seed in the right ACC, right SPL, and right SMG did not show any significant differences between correlation patterns for the patient compared to controls. Further, we did not see any significant voxels beyond the 4-SD range for the control seed placed in the left motor cortex.

These results indicate that (1) there is something special about the functional connectivity with the left MFG and that (2) a certain amount of reorganization may have occurred in the patient's brain to compensate for the missing tissue in the right MFG resulting in the paradoxical increase in activity coupling with the left MFG.

## Discussion

The current study elucidated the role of the right MFG as a gateway between top-down and bottom-up control of attention. By comparing performance on several attentional cueing tasks between a patient with a right MFG resection and a group of healthy controls, we uncovered how this region contributes to reorienting one's attention to stimuli in our environment.

### Role of right MFG in attentional reorienting

First, as expected, we found that our patient performed similar to controls on a Posner-type cueing task, i.e., he showed the same enhancement in accuracy and RT for stimuli presented at validly cued locations vs. invalid ones. This result confirms our hypothesis that the right MFG does not play a direct role in top-down attentional control, a function traditionally attributed to the dorsal attention network (DAN). Our result is therefore in line with what has previously been reported by many investigators about which brain regions process endogenously cued stimuli (e.g., Vossel et al., 2006; Mukai et al., 2011).

Second, contrary to our expectation, the patient showed no impairment relative to controls on exogenously cued trials that had a short ISI between cue and target stimulus. This result indicates that the right MFG may not play a direct role in exogenous attention either, a function typically thought to be controlled by the ventral attention network (VAN). Our result is therefore contrary to what has been reported previously about the role of the right MFG in processing exogenously cued stimuli (Corbetta et al., 2002; Fox et al., 2006). Critically, however, the patient exhibited difficulty on long ISI trials, indicating that he had trouble reverting to top-down attentional control, once the facilitatory effect of the exogenous cue had extinguished. The patient's performance on the simple and complex Go/No-Go tasks, eliminated the possibility of deficits in response inhibition or working memory as a potential reason for his abnormal behavior at long ISIs. Further, by explicitly cueing the patient during long ISI trials to attend to both stimulus locations (i.e., to explicitly divide attention between the two possible stimulus locations), we were able to force the patient to re-engage successfully in top-down control, resulting in a more normal behavioral profile. This result therefore indicates that the right MFG may play an important role in reorienting attention from exogenous to endogenous attentional control. Although a few studies have implicated the right MFG in mediating exogenous attention (Corbetta et al., 2002; Kincade et al., 2005; Fox et al., 2006), our results suggest that this node of the VAN would is mainly active when one reorients to top-down attention after an exogenous event.

In many studies of attention, researchers often use invalidly cued trials to understand how exogenous attention works. On such trials, the stimulus appears in an unexpected location away from the expected, cued location. Performance on these trials, when compared with neutral cue trials (uninformative cues appear at both locations), can provide an estimate of how subjects respond to an exogenous stimulus. However, we did not see a difference between the patient and control group in accuracy or RT on invalid trials relative to neutral cue trials. This finding further supports the notion that the right MFG is more involved in reorienting to top-down attention after the effects of an exogenous event have dissipated, and goes hand-in-hand with our findings in the explicit reorienting experiment.

One explanation for the discrepancy between our results and those of other researchers may be that our patient's resection was slightly anterior to the location of the right MFG node proposed by some investigators (Arrington et al., 2000; Corbetta et al., 2000; Mayer et al., 2004; Shulman et al., 2010). For example, Shulman et al. (2010) reported activity for exogenous shifts of attention in a right MFG region centered around Talairach coordinates: *x* = 45, *y* = 27, *z* = 24. This location is nearly 2 cm posterior to the location of our seed voxel in right MFG (Talairach coordinates: *x* = 31, *y* = 45, *z* = 26). Thus, it is entirely possible that the more posterior parts of the right MFG are indeed involved in processing of exogenous stimuli while more anterior regions (overlapping with our patient's resection) may be involved in engaging top-down attention after an exogenous cue has extinguished. Another possible reason for the discrepancy between our findings and those reportedly previously is that our patient's resection included not just right MFG but other parts of prefrontal cortex as well, such as areas in lateral prefrontal cortex (BA 9, 46, and 10). It is possible that these other regions, and not just the right MFG, play a role in processing of exogenous stimuli and reorienting of attention, and may have contributed to our result.

In addition to the deficits noted at longer ISIs, we found a surprising trend for the facilitation at 0 ms ISI to be smaller for the patient relative to controls. We hypothesize that since the patient showed a normal facilitation effect at 100 ms, it is possible that the tumor and subsequent resection of areas in right MFG may have introduced some latency/slowing in exogenous orienting, resulting in an inability to respond effectively to a stimulus that follows right at the heels of the cue (0 ms ISI). In contrast, for longer ISIs trials, this latency effect is diminished due to the temporal spacing of the cue and stimulus and exogenous orienting can proceed resulting in normal facilitation.

In all, the patient's behavior profiles looked very similar to controls on most tasks except for the long ISIs trials where he had a tendency to respond prematurely when the effect of the exogenous cue had dissipated. Based on our results we concluded that the patient was unable to revert to top-down attentional control resulting in his premature button presses. It is possible that during long ISIs the patient was in some “neutral state” between exogenous and endogenous modes of attention, and he initiated a button press because he was aware that he was supposed to do “something” but his attentional network was unable to keep track of what that “something” was.

### Changes in underlying circuitry in the damaged brain

Our resting state fMRI results indicated that changes had occurred in the patient's brain before or after tumor resection, such that the brain regions that are normally connected to the resected area significantly increased their coupling with the area's homolog in the left hemisphere. Regions that showed higher correlations relative to controls included two regions in the right hemisphere: the superior parietal lobule (SPL) and orbitofrontal cortex (OFC). The right SPL and neighboring regions in parietal cortex have been consistently associated with a critical role in visuospatial attention (Corbetta and Shulman, 2002; Yantis et al., 2002) and working memory (see meta-analysis by Owen et al., 2005). Thus, it is not surprising that, after resection of right MFG (including parts of dorsolateral prefrontal cortex (dlPFC; BA 9 and 46)—an area that is also implicated in attention (Rosen et al., 1999) and working memory (see Barbey et al., 2013 for review), its left hemisphere homolog showed enhanced connectivity with regions in the parietal lobe. This enhanced connection of the left MFG with right SPL might be a compensatory mechanism to reduce the impact of the missing tissue on attention and working memory. The right OFC has been previously implicated in decision-making (see Fellows, 2011; Rushworth et al., 2011 for reviews) and impulse control (Torregrossa et al., 2008). Thus, tighter coupling of the left MFG with this region is also not surprising given the role of BA 9 and 46 in these same cognitive functions, i.e., decision making (Heekeren et al., 2004; Fleck et al., 2006) and impulsivity (Garavan et al., 1999; Cho et al., 2010). Thus, in the absence of the right MFG, the left hemisphere homolog and its surrounding regions appear to have compensated (either during tumor growth or after resection) by increasing communication with other nodes of the visuospatial, decision making and impulse control circuitry.

These finding are consistent with several reports in the literature of compensatory changes in the human brain following injury. For example, in an fMRI study of recovery from acute spatial neglect in patients with right hemisphere stroke, Thimm et al. (2008) showed compensatory recruitment of areas in the contralesional frontoparietal attention network. In another fMRI study, Maruishi et al. (2007) showed compensatory activation of contralateral prefrontal regions in patients with diffuse axonal injury. Our findings are also compatible with a recent report about enhanced connectivity in resting state networks of the brain, as measured by MEG, after tumor resection (van Dellen et al., 2012). In fact, researchers in that study found that the increase in alpha band resting state network connectivity correlated well with the improved cognitive outcome after neurosurgical resection. In our study, the patient's performance on a host of neuropsychological tests indicated that, overall, his resection had not resulted in severe deficits in any cognitive domain. Indeed, only a slight psychomotor slowing was noted, as was perhaps some inattention as measured by the Wisconsin Card Sort Task and Continuous Performance Test. There was no evidence of spatial neglect as measured by his performance on the Behavioral Inattention Test, which included line bisection and article reading (classic tools for measuring hemispatial neglect). Thus, it is plausible that the enhanced communication that we saw in our patient within regions originally connected to the right MFG helped to diminish the impact of the lost tissue on cognitive function.

In conclusion, taken together our behavior and rsfMRI results suggest that the right middle frontal cortex plays a role in reorienting of attention, but that reorganization of the brain, either during tumor growth or after surgery, may help to diminish the impact of the lost tissue in this brain region.

### Conflict of interest statement

The authors declare that the research was conducted in the absence of any commercial or financial relationships that could be construed as a potential conflict of interest.
