# Supplementary figures and images for: A role of right middle frontal gyrus in reorienting of attention: a case study
Source: Front Syst Neurosci. 2015 Mar 3;9:23. doi: 10.3389/fnsys.2015.00023 (PMC4347607; doi:10.3389/fnsys.2015.00023)

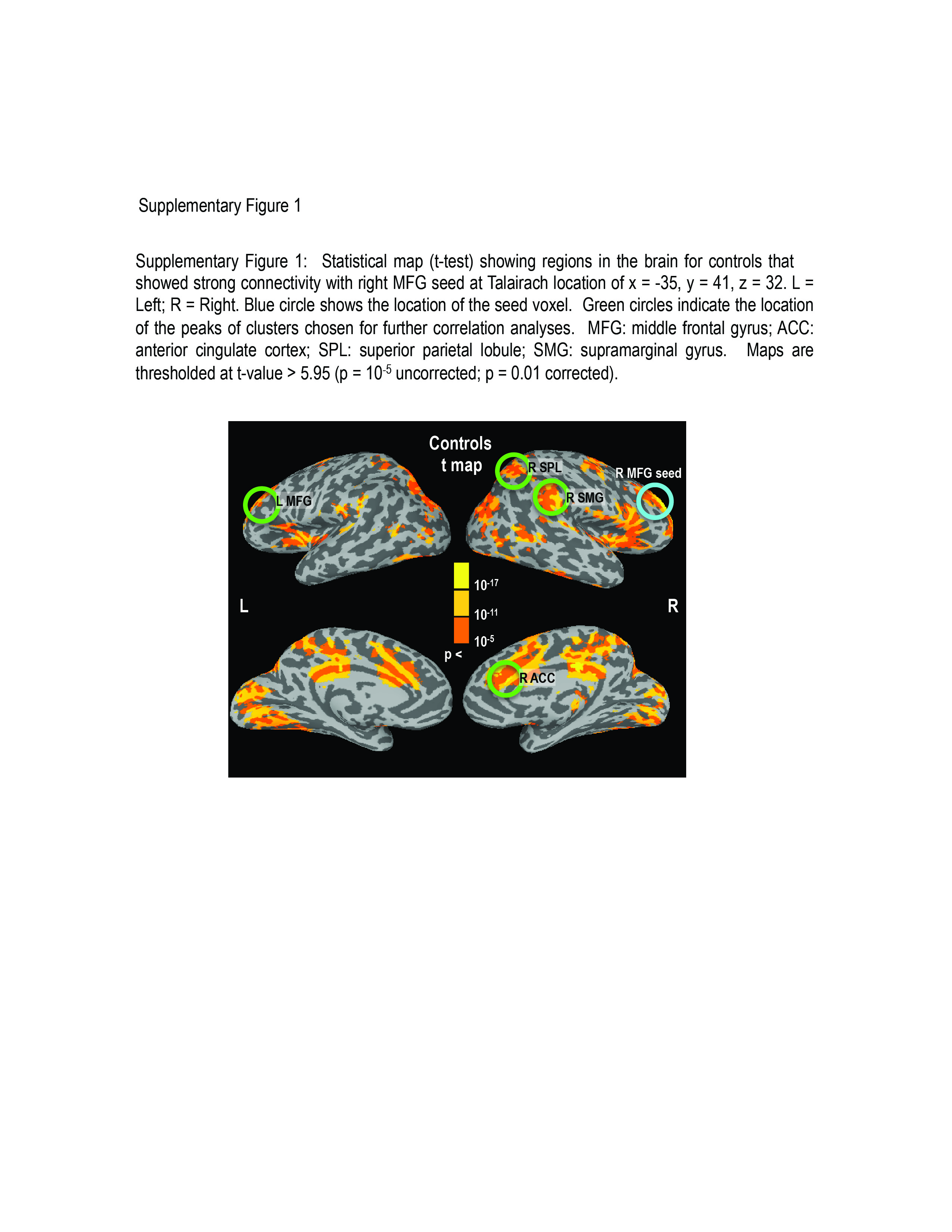

Supplement: Supplementary file 1 [file Image1.JPEG]

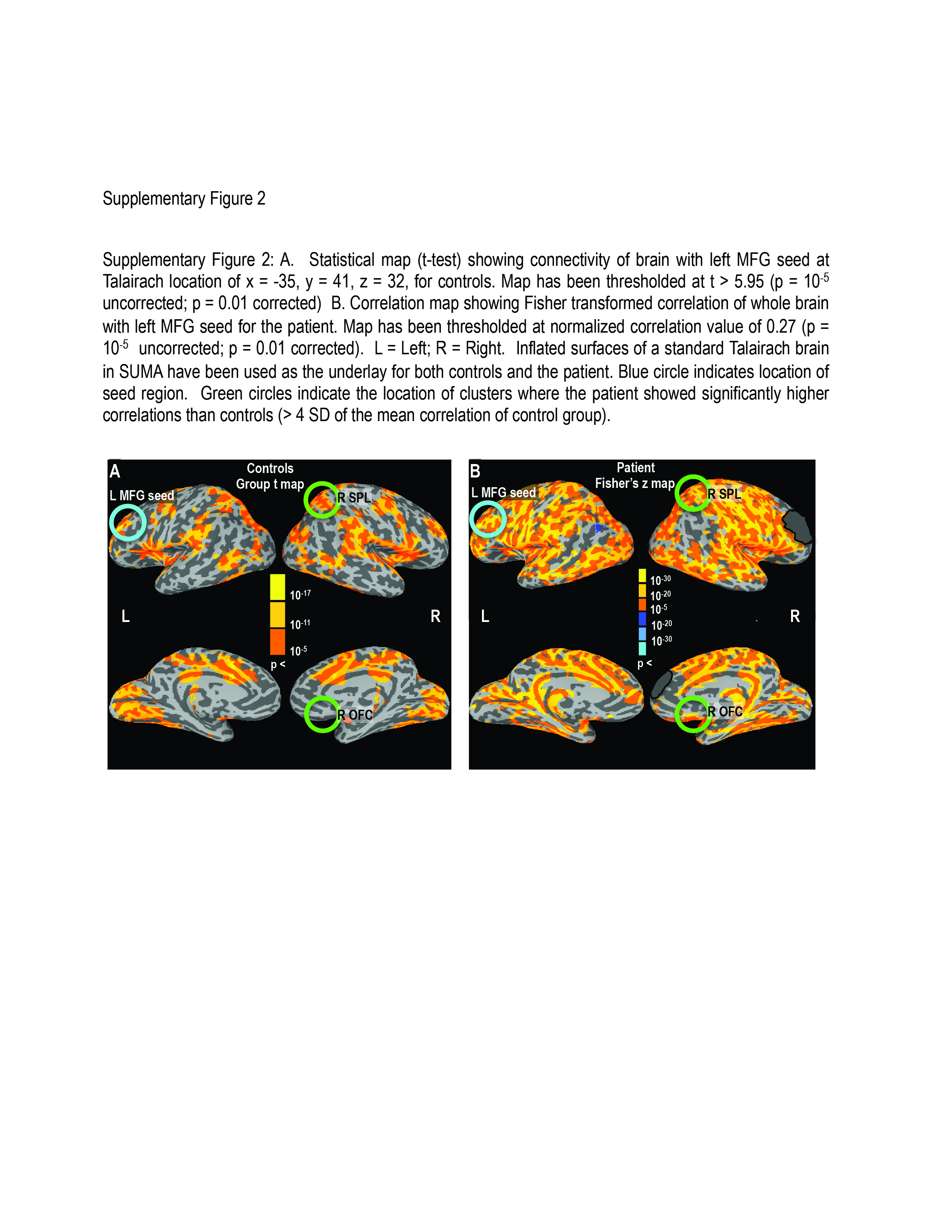

Supplement: Supplementary file 2 [file Image2.JPEG]
